# The long-term effects of multidrug immunosuppressive protocols based on calcineurin inhibitors and conversion to rapamycin on the morphology, apoptosis, and proliferation of rat salivary glands

**DOI:** 10.1007/s43440-023-00521-0

**Published:** 2023-09-02

**Authors:** Marta Grabowska, Karolina Kędzierska-Kapuza, Andrzej Kram, Kamil Gill, Leszek Teresiński, Olimpia Sipak, Małgorzata Piasecka

**Affiliations:** 1 Department of Histology and Developmental Biology, Faculty of Health Sciences, Pomeranian Medical University, Żołnierska 48, 71-210 Szczecin, Poland; 2 Department of Gastroenterological Surgery and Transplantation, Central Hospital of Ministry of Internal Affairs and Administration, Wołoska 137, 02-507 Warsaw, Poland; 3 Medical Center for Postgraduate Education, Warsaw, Poland; 4 Department of Pathology, West Pomeranian Oncology Center, Strzałowska 22, 71-730 Szczecin, Poland; 5 Department of Obstetrics and Pregnancy Pathology, Faculty of Health Sciences, Pomeranian Medical University, Żołnierska 48, 71-210 Szczecin, Poland

**Keywords:** Rapamycin, Calcineurin inhibitors, Apoptosis, Proliferation, Salivary glands, Collagen

## Abstract

**Background:**

The effect of multidrug immunosuppressive protocols on the salivary glands is still unknown. This study aimed to determine the influence of immunosuppressive regimens based on calcineurin inhibitors (CNIs) and conversion to rapamycin on the morphology, apoptosis, and proliferation of rat salivary glands.

**Methods:**

Male rats received cyclosporin A (CsA), tacrolimus (FK-506), mycophenolate mofetil (MMF), rapamycin (Rapa), and prednisone (Pre) according to three-drug protocols: CMP (CsA, MMF, and Pre), CMP/R (CsA, MMF, and Pre with conversion to Rapa), TMP (FK-506, MMF, and Pre), and TMP/R (FK-506, MMF, and Pre with conversion to Rapa). Morphological and immunohistochemical and quantitative analyses of the salivary glands were performed.

**Results:**

Structural changes in salivary glands were observed in all experimental groups, especially in the submandibular gland. In the salivary glands, the percentages of collagen fibers and TUNEL-, Ki67- and PCNA-positive cells were higher in the experimental groups vs. the control but were lower in the CMP/R and TMP/R groups vs. the CMP and TMP groups, with the exception of collagen fibers in the parotid gland in the TMP/R group vs. the TMP group.

**Conclusions:**

Long-term administration of CNIs in triple regimens and after conversion to rapamycin monotherapy, causes morphological changes in the salivary glands of rats. Immunosuppressive treatment based on CNIs is associated with an increase in collagen accumulation. The effects of the conversion of treatment with CNIs to rapamycin in immunosuppressive protocols in rat salivary glands lead to decreased fibrosis, apoptosis, and proliferation. These changes may possibly prevent abnormalities resulting from the application of CNIs.

**Supplementary Information:**

The online version contains supplementary material available at 10.1007/s43440-023-00521-0.

## Introduction

Long-term use of immunosuppressive drugs is common in vascularized organ transplant patients, as well as in inflammatory and autoimmune diseases. In transplant recipients, these drugs are used to prevent immune-borne rejection of transplanted organs, which is associated with increased patient survival. Transplant rejection is a complex process influenced by both immunological and nonimmunological factors. It is important to choose the proper immunosuppressive treatment protocol, which will have a low rate of side effects and significantly improve the quality of life of patients [1–3].

In transplant recipients, the standard triple medication protocol includes a combination of calcineurin inhibitors (CNIs), including cyclosporine A (CsA) and tacrolimus (FK-506), an antiproliferative agent, e.g., mycophenolate mofetil (MMF), a corticosteroid, e.g., prednisone, and less mammalian target of rapamycin (mTOR) inhibitors, such as rapamycin (sirolimus) [2]. The use of multidrug protocols involving drugs characterized by different mechanisms of action and non-overlapping toxicity profiles and/or therapeutic pathways allows modulation of the immune response and a reduction in side effects by lowering the doses of individual drugs. Accordingly, immunosuppressive drugs may mediate their effects on one or more cell types, thereby reducing inflammation and immune responses [2, 3].

CNI-based immunosuppressive treatment protocols are important in maintaining the proper function of the graft, but they have a narrow therapeutic window and show a number of side effects. The long-term use of CNIs is associated with nephrotoxicity that may contribute to chronic graft dysfunction. However, it has been shown that rapamycin, showing strong immunosuppressive and antiproliferative effects and high efficiency in preventing acute rejection of transplants, may act synergistically with CsA, inhibiting the proliferation of activated lymphocytes. Moreover, rapamycin has a favorable nephrotoxicity profile compared to CNIs. It has also been suggested that multidrug therapy with rapamycin may be associated with delayed drug resistance [4–6]. It is worth noting that the antiproliferative properties of rapamycin may be associated with a lower risk of malignancy in organ transplant patients. Rapamycin has been shown to affect angiogenesis as well as cell growth, division and survival. Therefore, in clinical practice, patients diagnosed with cancer are converted from treatment based on CNIs to monotherapy with the use of a drug from the group of mTOR inhibitors. These drugs can be included in therapy both a few months after transplantation and many years after this procedure. In addition, treatment regimens based on mTOR kinase inhibitors are also used in patients with polyoma BK virus and cytomegalovirus infection [7–9].

The available literature provides information on the known side effects of CsA and FK-506 in the form of gingival overgrowth and alveolar bone loss [10, 11]. In addition, immunosuppressants have been shown to be associated with an increased risk of oral infections in the form of erythematous candidiasis, hairy leukoplakia or saburral tongue [12, 13]. It has also been revealed that within the oral cavity, CNIs can induce xerostomia (dry mouth) or hyposalivation. Due to the high lipophilicity of CsA, it is transferred from the blood to the saliva and can cause changes in the composition of the saliva. As a result, patients are at increased risk of oral infections, caries, and changes in taste, which may consequently lead to a decrease in quality of life [14–17].

There are very few scientific reports on the effect of immunosuppressive drugs on the morphology of salivary glands [16–18]. Unfortunately, these reports show only the influence of single immunosuppressants on these glands. The authors have revealed that treatment with either CsA or FK-506 is associated with significant changes in the saliva composition and the morphology of the parotid and submandibular glands in rats [16, 17]. To date, the long-term effects of CNIs in multidrug protocols and conversion from CNIs to rapamycin on the salivary glands are unknown, although currently, immunosuppressive therapy in patients after organ transplantation is usually based on multidrug protocols. Therefore, this study was designed to determine the long-term influence of three-drug protocols of immunosuppressive treatment used in clinical practice based on CNIs and conversion of these treatments to monotherapy with rapamycin on the morphology, collagen accumulation and apoptosis and proliferation in the rat parotid, submandibular and sublingual glands.

## Materials and methods

### Animals

The experiment was conducted on 30 3-month-old male Wistar rats. The sexually mature animals were obtained from a licensed breeder from the Institute of Occupational Medicine (Lodz, Poland) and had certificates issued by an official veterinarian confirming their health and genetic status. The rats were adapted to the experiment for 2 weeks. The animals were typically housed in simple cages consisting of one open space (*n* = 6 per cage), had free access to water throughout the experiment and received a high-quality specialized laboratory diet of LSM type (Agropol Motycz, Lublin, Poland). The housing temperature was maintained at a humidity of 50 ± 5% and 21 °C, and the dark:light cycle was set at 12-h light:12-h dark. The initial body mass of the animals was approximately 305 g.

The animals were randomly divided into five groups (*n* = 6 in each group): the control group and four experimental groups (CMP, CMP/R, TMP, and TMP/R). The rats in the control group were administered just bread balls vehicle with no added drugs. In the experimental groups rats received orally the following immunosuppressive drugs: CsA (Sandimmum-Neoral, Novartis International, Basel, Switzerland), FK-506 (Prograf, Astellas Pharma, Tokyo, Japan), MMF (CellCept, Hoffman-La Roche, Basel, Switzerland), prednisone (Encorton, Polfa, Pabianice, Poland), and rapamycin (Rapamune, Pfizer, New York, NY, USA), according to the three-drug protocols used in patients after organ transplantation. The following daily drug doses were used: CsA—5.0 mg/kg of body weight, FK-506—4.0 mg/kg of body weight, MMF—20.0 mg/kg of body weight, prednisone—4.0 mg/kg of body weight, and rapamycin—0.5 mg/kg of body weight. In the CMP group, rats received CsA, MMF, and prednisone; in the CMP/R group, rats received CsA, MMF, and prednisone in the first 3 months and rapamycin in the last 3 months; in the TMP group, rats received FK-506, MMF, and prednisone; and in the TMP/R group, rats received FK-506, MMF, and prednisone in the first 3 months and rapamycin in the last 3 months. The animals received drugs using their pharmaceutical form in bread balls every day for 6 months. To obtain reliable results, the drug doses were adjusted to the body mass of rats and properly calculated considering the metabolic differences between rats and humans. The drug concentrations in the blood of the rats were within the therapeutic range as previously published by Grabowska et al. [19].

This study was conducted by the approval of the Local Commission of Ethics for the Care and Use of Laboratory Animals (No. 26/2011 of 16th December 2011, Pomeranian Medical University in Szczecin, Poland).

### Collection of the salivary glands

Rats were anesthetized intraperitoneally with 50 mg/kg ketamine hydrochloride. During sectioning, the parotid, submandibular and sublingual salivary glands of rats were obtained. Salivary glands of the control and experimental groups were fixed in 4% paraformaldehyde in phosphate buffer for 24 h at 4 °C and embedded into paraffin. The paraffin blocks were cut into 3-μm sections, which were placed on polylysine-coated slides for further analysis.

### Morphological studies

Sections of the parotid, submandibular, and sublingual salivary glands were deparaffinized using xylene followed by hydration in a series of graded alcohols. Next, the sections were stained using standard methods: hematoxylin and eosin (HE) for routine histological examination and Masson trichrome to reveal the collagen fibers.

### TUNEL assay

Terminal deoxynucleotidyl transferase dUTP nick-end labeling (TUNEL) assay was carried out according to the manufacturer’s instructions (S7100; ApopTag Peroxidase In Situ Apoptosis Detection Kit; Millipore, Billerica, MA, USA) previously described in detail by Grabowska et al. [20]. The negative controls for reaction specificity were performed by replacing the reaction mixture containing terminal deoxynucleotidyl transferase (TdT; Millipore, Billerica, MA, USA) with label solution only (without TdT). The sections were examined using a light microscope (Olympus BX 41, Hamburg, Germany).

### Immunohistochemistry

The immunostaining procedure was performed according to the protocol previously described in detail by Grabowska et al. [19]. To reveal antigens, the salivary gland sections were boiled in Target Retrieval Solution (S2368; Dako, Glostrup, Denmark) at pH 9.0 for 30 min. The quenching endogenous peroxidase activity in tissue sections was carried out by using a peroxidase-blocking solution (S2023; Dako, Glostrup, Denmark) for 10 min. To determine the immunolocalization and immunoexpression of Ki67, the mouse monoclonal anti-Ki67 antigen–antibody (IR626; clone MIB-1; Dako, Glostrup, Denmark; 1:100), and the mouse monoclonal anti-proliferating cell nuclear antigen (PCNA)-antibody (sc-25280; clone F-2, Santa Cruz Biotechnology, Dallas, TX, USA; 1:250) was used. The specificity of immunostaining was confirmed by following the above procedures by replacing the primary antibody with IgG from mouse and rabbit serum, respectively.

### Quantitative analysis of collagen and immunohistochemistry

Masson trichrome-stained and also TUNEL-, Ki67-, and PCNA-immunostained slides were scanned at a magnification of 400 × on the Aperio AT2 digital slide scanner (Leica Microsystems, Wetzlar, Germany). The scanning resolution was 0.25 μm/pixel. The background illumination levels were calibrated using a prescan procedure. Moreover, the scanner was configured to minimize focus problems. The obtained digital slides of the tissues were examined on the computer screen using the ImageScope viewer (Version 11.2.0.780; Aperio Technologies, Vista, CA, USA).

For the quantitative analysis of collagen fibers in rat salivary glands stained with Masson trichrome, a positive pixel count v9 algorithm (version 9.1; Aperio Technologies, Vista, CA, USA) was used. Other parameters were set to achieve compliance with the visual evaluation. The areas of analysis were manually determined. The percentage of collagen fibers that were positive for Masson's trichrome staining was calculated in 30 fields in each group.

For the quantitative analysis of TUNEL-, Ki67-, and PCNA-positive cells in the salivary glands, a nuclear v9 algorithm (version 9.1; Aperio Technologies, Vista, CA, USA) was used. Other parameters were set to achieve compliance with the visual evaluation, taking into account the threshold for a positive result, a brown color of the reaction in the cell nucleus. The areas of analysis were also manually determined. Using the algorithm, the percentage of positive nuclei was determined. The total number of positive nuclei was counted in 30 fields in each group (five high-power fields from each rat (*n* = 6) with an average analysis area not less than 0,96 mm^2^).

### Statistical analysis

All statistical analyzes were performed by using TIBCO Statistica version 13.3 (TIBCO Software, Palo Alto, CA, USA). The medians and ranges were calculated. Since the obtained values failed the normal distribution assumption and the groups of animals were small (*n* = 6), the non-parametric Kruskal–Wallis test followed by Dunn’s multiple comparison test for post hoc analysis was used to assess the differences between the groups. The probability *p* ≤ 0.05 was considered statistically significant.

## Results

### Morphological studies

#### Parotid salivary gland

**Fig. 1:**
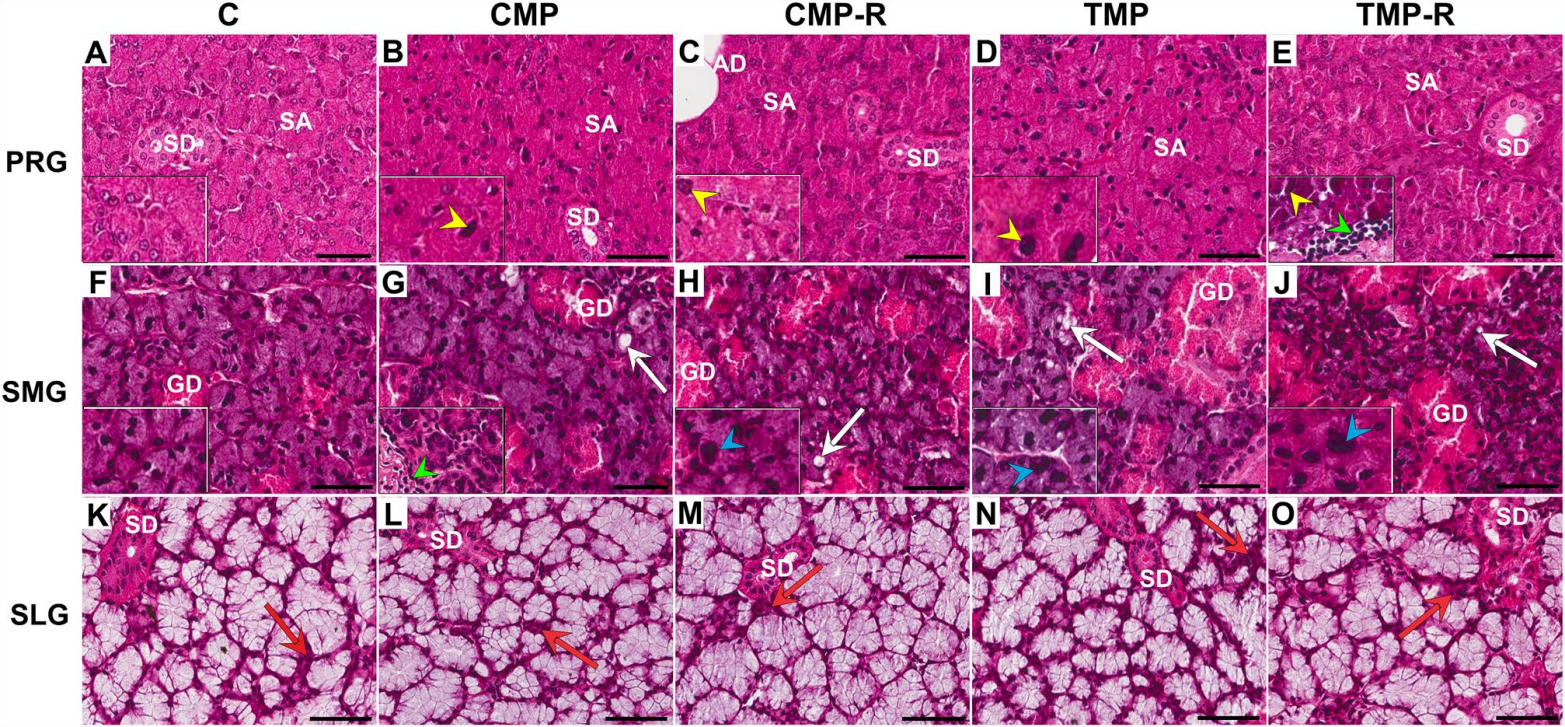
Representative light micrographs of hematoxylin and eosin-stained parotid (**A**–**E**), submandibular (**F**–**J**) and sublingual glands (**K**–**O**) of rats in the control (**A**, **F**, **K**), CMP (**B**, **G**, **L**), CMP/R (**C**, **H**, **M**), TMP (**D**, **I**, **N**) and TMP/R (**E**, **J**, **O**) groups. Parotid gland (PRG): normal serous acini (A), morphological changes in the form of cells with an increased nucleus to cytoplasm ratio (yellow arrowheads) (**B**–**E**), and focal infiltrations of inflammatory cells (green arrowhead) (**E**). Submandibular salivary gland (SMG): normal basophilic acinar cells and granular ducts (**F**), cells with acinar vacuolization (white arrows) (**G**–**J**), inflammatory cell infiltrates (green arrowhead) (**G**), and atypical hyperchromatic and polymorphic nuclei (blue arrowheads) (**H**–**J**). Sublingual gland (SLG): typical mucous acini surrounded by few serous acini forming the serous demilunes (red arrows) (**K**–**O**). *AD* adipocyte; *C* control group without any medication; *CMP* rats treated with cyclosporin A, mycophenolate mofetil, and prednisone; *CMP/R* rats treated with cyclosporin A, mycophenolate mofetil, and prednisone in the first 3 months of the experiment and rapamycin in the last 3 months; *GD* granular duct; *SA* serous acini; *SD* striated duct; *TMP* rats treated with tacrolimus, mycophenolate mofetil, and prednisone; *TMP/R* rats treated with tacrolimus, mycophenolate mofetil, and prednisone in the first 3 months of the experiment and rapamycin in the last 3 months. Scale bar—50 µm

**Fig. 2:**
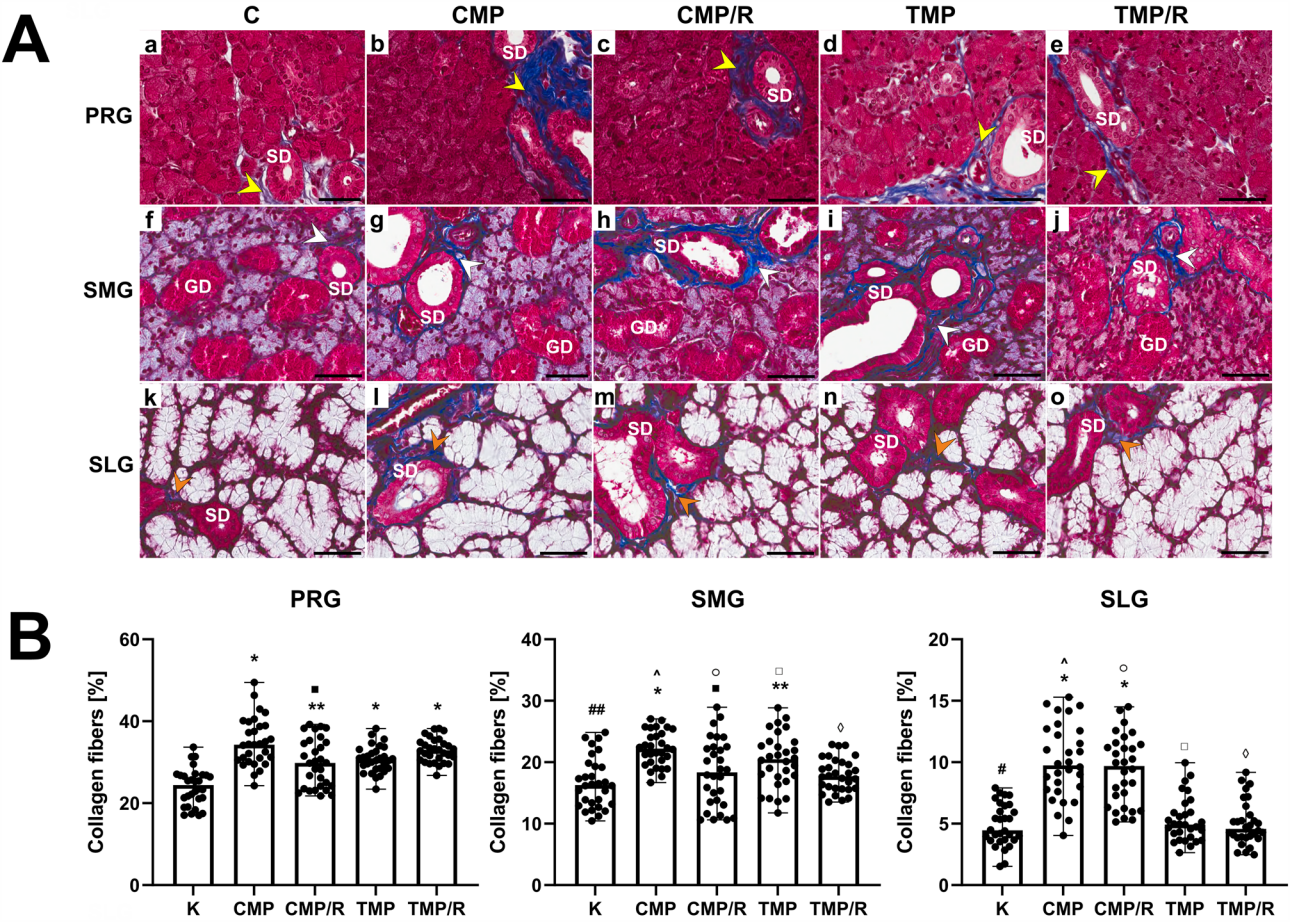
Effect of multidrug immunosuppressive protocols based on calcineurin inhibitors and conversion to rapamycin on the collagen deposition in rat salivary glands. **A** Representative light micrographs of Masson trichome-stained (blue-stained collagen fibers) parotid (a–e), submandibular (f–j) and sublingual glands (k–o) of rats in the control (a, f, k), CMP (b, g, l), CMP/R (c, h, m), TMP (d, i, n) and TMP/R (e, j, o) groups. Normal distribution of collagen fibers (yellow, white and orange arrowheads, respectively) in the control groups (a, f, k). Parotid gland (PRG): visible interstitial fibrosis—increased deposition of collagen fibers (yellow arrowheads) (b–e). Submandibular gland (SMG) and sublingual gland (SLG): slight deposition of collagen fibers (white arrowheads) (g–j), (orange arrowheads) (l–o), respectively. Scale bar—50 µm. **B** Comparison of the percentages of collagen in the parotid, submandibular and sublingual glands of rats between the control and experimental groups. Results are shown as medians and ranges, *n* = 6 rats in each group. In individual salivary glands: *p < 0.001 vs. control; **p < 0.05 vs. control; ^■^*p* < 0.05 vs. CMP. Between the salivary glands: ^#^*p* < 0.001 vs. control in parotid gland; ^##^*p* < 0.05 vs. control in parotid gland; ^*p* < 0.001 vs. CMP in parotid gland; ^○^*p* < 0.001 vs. CMP/R in parotid gland; ^□^*p* < 0.001 vs. TMP in parotid gland; ^◊^*p* < 0.001 vs. TMP/R in parotid gland (Kruskal–Wallis test followed by a Dunn’s multiple comparison post hoc test). *C* control group without any medication; *CMP* rats treated with cyclosporin A, mycophenolate mofetil, and prednisone; *CMP/R* rats treated with cyclosporin A, mycophenolate mofetil, and prednisone in the first 3 months of the experiment and rapamycin in the last 3 months; *GD* granular duct; *PRG* parotid gland; *SD* striated duct; *SLG* sublingual gland; *SMG* submandibular gland; *TMP* rats treated with tacrolimus, mycophenolate mofetil, and prednisone; *TMP/R* rats treated with tacrolimus, mycophenolate mofetil, and prednisone in the first 3 months of the experiment and rapamycin in the last 3 months

In the rat parotid glands from the control and experimental groups, typical protein-secreting serous acini were observed. The acinar serous cells were predominantly columnar or pyramidal with basally located nuclei and were characterized by indistinct cellular borders. In the basal and perinuclear regions, basophilic cytoplasm was usually noted, whereas in the apical region, more eosinophilic cytoplasm containing numerous zymogen granules was revealed. The intercalated ducts were lined by cuboidal epithelium. In turn, the striated ducts (showing vertical striations) and interlobular excretory ducts were lined with columnar epithelial cells. Moreover, in the parotid gland, adipose tissue often occurred (Fig. 1A–E, Supplementary Fig. 1). However, in all experimental groups (CMP, CMP/R, TMP, and TMP/R), structural changes were found. In a few areas, cells with an increased nucleus-to-cytoplasm ratio, enlarged nuclei with irregular shapes, and hyperchromasia were noted (Fig. 1B–E, Supplementary Fig. 1). Furthermore, focal infiltrations of inflammatory cells in the TMP/R group were also observed (Fig. 1E). Interstitial fibrosis associated with the accumulation of collagen fibers was observed in all experimental groups. The collagen fibers were visible predominantly in the connective tissue septa between the lobules surrounding intralobular and large excretory ducts and blood vessels (Fig. 2Ab–e, Supplementary Fig. 2).

#### Submandibular salivary gland

In rat submandibular glands from the control and experimental groups, typical tubuloalveolar mixed protein-secreting serous acini and mucin-secreting mucous acini with a predominance of serous acini were noted. Very few scattered mucous acini within the main serous portion of the gland were observed. The acinar cells were mainly basophilic. The mucous acini were characterized by polyhedral cells with flattened and basally located nuclei. The serous acini were similar to those in the parotid gland. The granular ducts, characteristic of rodents, were lined by cuboidal or columnar epithelial cells with eosinophilic secretory granules in the cytoplasm. The intercalated ducts were lined by cuboidal epithelial cells, while striated ducts and interlobular excretory ducts were lined with columnar epithelial cells with nuclei located in the central or apical region. It is worth mentioning that within the submandibular gland, no adipose tissue was noticed compared to the parotid salivary gland (Fig. 1F–J, Supplementary Fig. 1). In turn, in all experimental groups, especially in the CMP/R and TMP/R groups, more advanced structural changes were observed in some areas of the gland compared to the parotid and sublingual salivary glands. Cells with a clear disturbance of the nucleus-cytoplasm ratio and with atypical hyperchromatic and polymorphic nuclei, sometimes with inclusions, were noticed. Intracytoplasmic vacuolization of acini, indicating atrophy of the secretory portion, was also observed (Fig. 1G–J, Supplementary Fig. 1). Furthermore, in the CMP and TMP groups, hypertrophied acini and numerous focal infiltrations of inflammatory cells were observed. Inflammatory cells in the vicinity of blood vessels and ducts and within the interstitial tissue were found (Fig. 1H, J). In all experimental groups, deposition of collagen fibers in the connective tissue septa surrounding intralobular and large excretory ducts was observed (Fig. 2Ag–j, Supplementary Fig. 2).

#### Sublingual salivary gland

In the rat sublingual glands from the control and experimental groups, normal tubuloalveolar mixed protein-secreting serous acini with a clear predominance of mucin-secreting mucous acini were found. In most terminal secretory units, typical mucous acini surrounded by a few serous acini forming serous demilunes were observed. The intercalated ducts were lined by cuboidal epithelium, whereas the interlobular excretory ducts were lined with columnar epithelial cells. In addition, within the sublingual gland, no adipose tissue was observed (Fig. 1K–O, Supplementary Fig. 1). In all experimental groups, slight structural changes were found. In very few areas, acinar nuclei were enlarged. Moreover, in the CMP and TMP groups, acini appeared reduced in size. In a few areas, a slight accumulation of collagen fibers in the connective tissue septa surrounding intralobular and large excretory ducts was observed (Fig. 2Al–o, Supplementary Fig. 2).

### Collagen percentages

Kruskal–Wallis and post hoc Dunn’s tests revealed that in the parotid gland, the percentage of collagen was significantly higher than that in the submandibular and sublingual glands for the control (*H*(2) = 70.63; *p* = 0.002 and *p* < 0.001, respectively), CMP (*H*(2) = 78.25; *p* < 0.001, respectively), CMP/R (*H*(2) = 67.62; *p* < 0.001, respectively), TMP (*H*(2) = 77.57; *p* < 0.001, respectively), and TMP/R (*H*(2) = 79.12; *p* < 0.001, respectively) groups. In turn, in the sublingual gland, the above parameter was significantly lower (*p* < 0.001, respectively) than that in the submandibular gland for all groups (Fig. 2B).

In the parotid gland, the obtained percentages of collagen (*n* = 30 in each group) were significantly higher in all experimental groups (*H*(4) = 55.70; *p* < 0.001 and *p* = 0.002, respectively) than in the control group. In the submandibular gland, the percentage of collagen (*n* = 30 in each group) was significantly higher only in the CMP and TMP groups (*H*(4) = 30.26; *p* < 0.001 and *p* = 0.018, respectively) vs. control. In turn, in the sublingual gland, the percentage of collagen (*n* = 30 in each group) was significantly higher in the CMP and CMP/R (*H*(4) = 73.64; *p* < 0.001, respectively) groups vs. control. Moreover, in the parotid and submandibular glands in the CMP/R group, the percentage of collagen was significantly lower than that in the CMP group (*p* = 0.022 and *p* = 0.006, respectively), while in the sublingual gland, no significant differences were noted. In all salivary glands in the TMP/R group, the above parameters were statistically nonsignificant vs. the TMP group. In addition, when comparing the percentage of collagen between the CMP and TMP groups, a lower percentage of collagen fibers was observed in the TMP group than in the CMP group, but the difference was statistically insignificant in all salivary glands (Fig. 2B).

### Immunolocalization and the percentage of TUNEL-positive cells

**Fig. 3:**
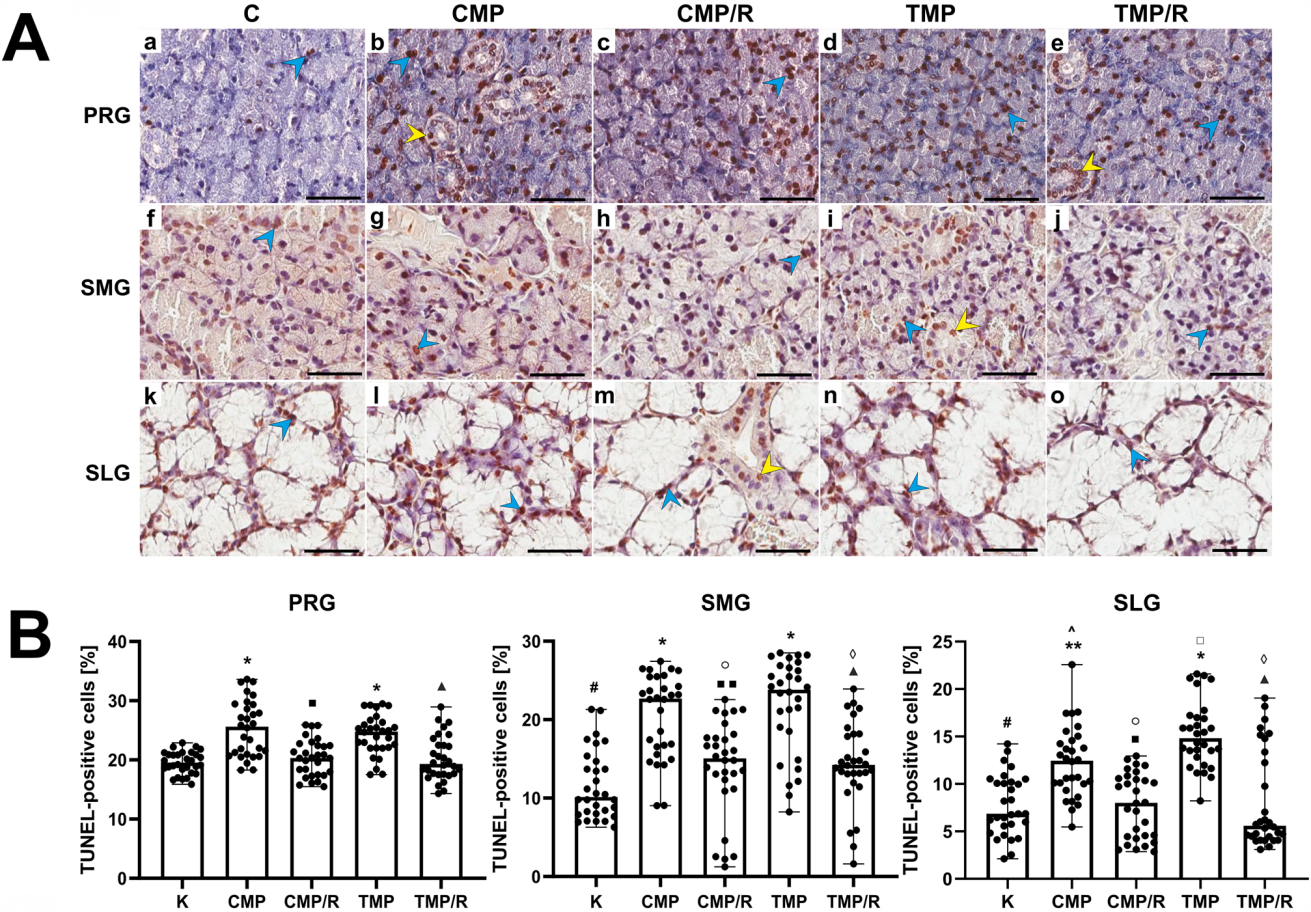
Effect of multidrug immunosuppressive protocols based on calcineurin inhibitors and conversion to rapamycin on the apoptosis in rat salivary glands.** A** Representative light micrographs of the histochemical reaction showing TUNEL-positive cells in the parotid (a–e), submandibular (f–j) and sublingual glands (k–o) of rats in the control (a, f, k), CMP (b, g, l), CMP/R (c, h, m), TMP (d, i, n) and TMP/R (e, j, o) groups. TUNEL-positive cells (brown-stained nuclei) in acini (blue arrowheads) and ducts (yellow arrowheads). Scale bar—50 µm. **B** Comparison of the percentages of TUNEL-positive cells in the parotid, submandibular and sublingual glands of rats between the control and experimental groups. Results are shown as medians and ranges, *n* = 6 rats in each group. In individual salivary glands: *p < 0.001 vs. control; **p < 0.05 vs. control; ^■^*p* < 0.001 vs. CMP; ^■■^*p* < 0.05 vs. CMP; ^▲^*p* < 0.001 vs. TMP. Between the salivary glands: ^#^*p* < 0.001 vs. control in parotid gland; ^*p* < 0.001 vs. CMP in parotid gland; ^○^*p* < 0.001 vs. CMP/R in parotid gland; ^□^*p* < 0.001 vs. TMP in parotid gland; ^◊^*p* < 0.001 vs. TMP/R in parotid gland (Kruskal–Wallis test followed by a Dunn’s multiple comparison post hoc test). *C* control group without any medication; *CMP* rats treated with cyclosporin A, mycophenolate mofetil, and prednisone; *CMP/R* rats treated with cyclosporin A, mycophenolate mofetil, and prednisone in the first 3 months of the experiment and rapamycin in the last 3 months; *PRG* parotid gland; *SLG* sublingual gland; *SMG* submandibular gland; *TMP* rats treated with tacrolimus, mycophenolate mofetil, and prednisone; *TMP/R* rats treated with tacrolimus, mycophenolate mofetil, and prednisone in the first 3 months of the experiment and rapamycin in the last 3 months; *TUNEL* terminal deoxynucleotidyl transferase-mediated dUTP nick end-labeling

In the control group and in the experimental groups of rats, TUNEL-positive cells (with nuclear DNA fragmentation) in the parotid, submandibular and sublingual glands were characterized by brown-stained nuclei of acini and ducts (Fig. 3A, Supplementary Fig. 3). Kruskal–Wallis and post hoc Dunn’s tests showed that in the parotid gland, the percentage of TUNEL-positive cells was significantly higher than that in the submandibular gland for the control (*H*(2) = 57.23; *p* < 0.001), CMP/R (*H*(2) = 54.65, *p* = 0.001), and TMP/R (*H*(2) = 45.01; *p* < 0.001) groups and was higher than that in the sublingual gland for the control (*H*(2) = 57.23; *p* < 0.001), CMP (*H*(2) = 50.89; *p* < 0.001), CMP/R (*H*(2) = 54.65; *p* < 0.001), TMP (*H*(2) = 37.92; *p* < 0.001), and TMP/R (*H*(2) = 45.01; *p* < 0.001) groups. In the sublingual gland, the percentage of these cells was significantly lower (*p* = 0.028, *p* < 0.001, *p* = 0.001, *p* = 0.011, respectively) than that in the submandibular gland for all groups (Fig. 3B).

The percentage of TUNEL-positive cells (*n* = 30 in each group) in the CMP and TMP groups was significantly higher in the parotid (*H*(4) = 44.19; *p* < 0.001, respectively), submandibular (*H*(4) = 53.70; *p* < 0.001, respectively) and sublingual (*H*(4) = 57.52; *p* = 0.002 and *p* < 0.001, respectively) glands than that in the control group. In all studied glands in the CMP/R and TMP/R groups, the percentage of TUNEL-positive cells was significantly lower than that in the CMP (*p* < 0.001 and *p* = 0.003, respectively) and TMP (*p* < 0.001) groups (Fig. 3B).

### Immunolocalization and the percentage of Ki67-positive cells

**Fig. 4:**
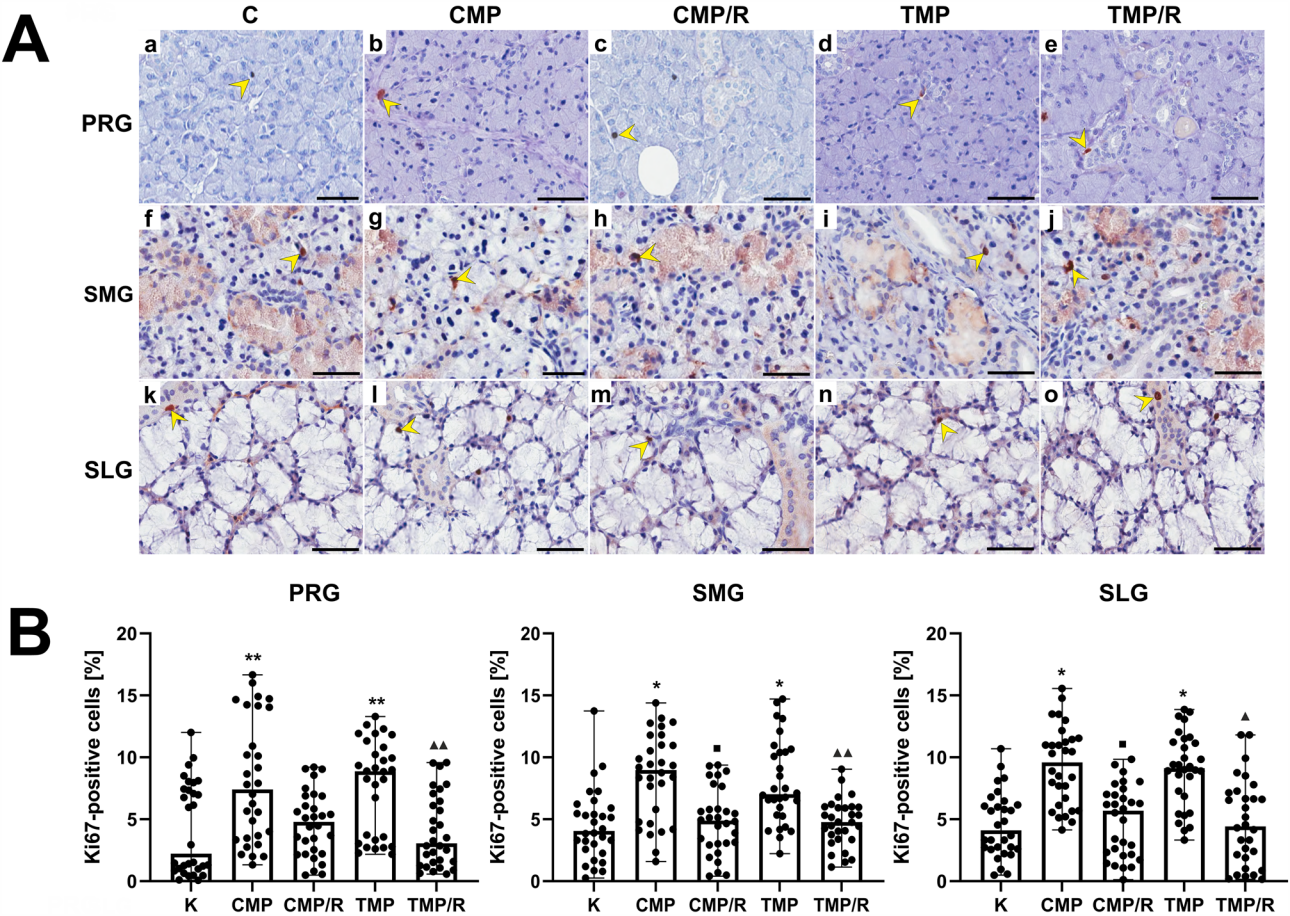
Effect of multidrug immunosuppressive protocols based on calcineurin inhibitors and conversion to rapamycin on the immunoexpression of Ki-67 in rat salivary glands.** A** Representative light micrographs of the immunohistochemical reaction showing Ki67-positive cells in the parotid (a–e), submandibular (f–j) and sublingual glands (k–o) of rats in the control (a, f, k), CMP (b, g, l), CMP/R (c, h, m), TMP (d, i, n) and TMP/R (e, j, o) groups. Ki67-positive cells were characterized by brown-stained nuclei (yellow arrowheads). Scale bar—50 µm. **B** Comparison of the percentages of Ki67-positive cells in the parotid, submandibular and sublingual glands of rats between the control and experimental groups. Results are shown as medians and ranges, *n* = 6 rats in each group. In individual salivary glands: *p < 0.001 vs. control; **p < 0.05 vs. control; ^■^*p* < 0.001 vs. CMP; ^▲^*p* < 0.001 vs. TMP; ^▲▲^*p* < 0.05 vs. TMP. (Kruskal–Wallis test followed by a Dunn’s multiple comparison post hoc test). *C* control group without any medication; *CMP* rats treated with cyclosporin A, mycophenolate mofetil, and prednisone; *CMP/R* rats treated with cyclosporin A, mycophenolate mofetil, and prednisone in the first 3 months of the experiment and rapamycin in the last 3 months; *PRG* parotid gland; *SLG* sublingual gland; *SMG* submandibular gland; *TMP* rats treated with tacrolimus, mycophenolate mofetil, and prednisone; *TMP/R* rats treated with tacrolimus, mycophenolate mofetil, and prednisone in the first 3 months of the experiment and rapamycin in the last 3 months

In the control group and in the experimental groups of rats, in all examined salivary glands, Ki67 immunolocalization in the form of brown-stained cell nuclei of acini and ducts was observed (Fig. 4A, Supplementary Fig. 4). Kruskal–Wallis and post hoc Dunn’s tests indicated that in the parotid gland, the percentage of Ki67-positive cells was statistically nonsignificant vs. that in the submandibular and sublingual glands for all groups. In the sublingual gland, there were no statistically significant differences in the percentage of these cells vs. the submandibular gland for all groups (Fig. 4B).

The percentage of Ki67-positive cells (*n* = 30 in each group) in the CMP and TMP groups was significantly higher in the parotid (*H*(4) = 27.01; *p* = 0.006 and *p* = 0.002, respectively), submandibular (*H*(4) = 36.15; *p* < 0.001 and *p* = 0.001, respectively) and sublingual (*H*(4) = 48.02; *p* < 0.001, respectively) glands than that in the control group. In the submandibular and sublingual glands in the CMP/R and TMP/R groups, the percentage of Ki67-positive cells was significantly lower than that in the CMP (*p* = 0.001 and *p* < 0.001, respectively) and TMP (*p* = 0.004 and *p* < 0.001, respectively) groups. In the parotid gland, this parameter was significantly lower (*p* = 0.002) only in the TMP/R group vs. the TMP group (Fig. 4B).

### Immunolocalization and the percentage of PCNA-positive cells

**Fig. 5:**
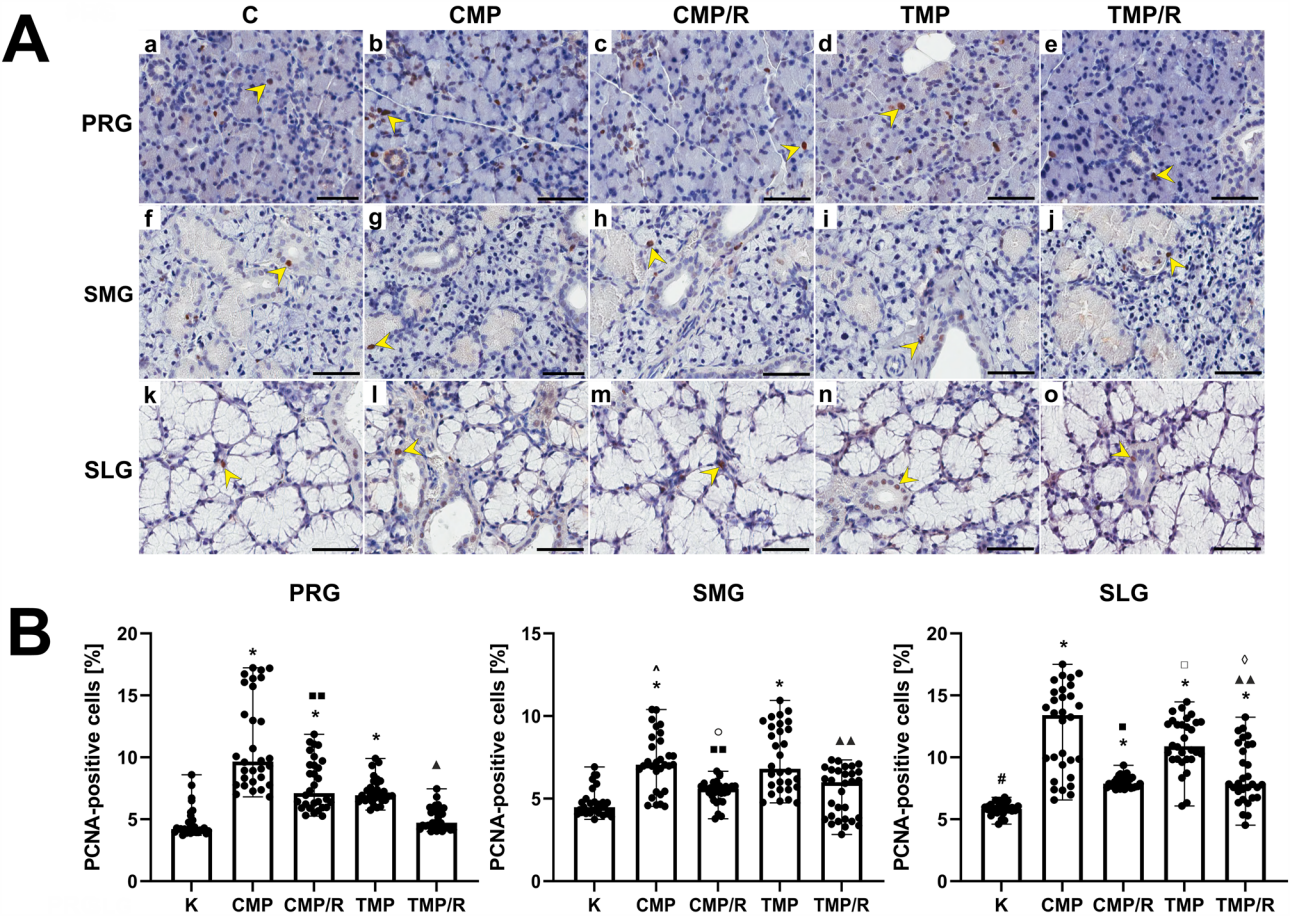
Effect of multidrug immunosuppressive protocols based on calcineurin inhibitors and conversion to rapamycin on the immunoexpression of PCNA in rat salivary glands. **A** Representative light micrographs of the immunohistochemical reaction showing PCNA-positive cells in the parotid (a–e), submandibular (f–j) and sublingual glands (k–o) of rats in the control (a, f, k), CMP (b, g, l), CMP/R (c, h, m), TMP (d, i, n) and TMP/R (e, j, o) groups. PCNA-positive cells were characterized by brown-stained nuclei (yellow arrowheads). Scale bar—50 µm. **B** Comparison of the percentages of PCNA-positive cells in the parotid, submandibular and sublingual glands of rats between the control and experimental groups. Results are shown as medians and ranges, *n* = 6 rats in each group. In individual salivary glands: *p < 0.001 vs. control; ^■^*p* < 0.001 vs. CMP; ^■■^*p* < 0.05 vs. CMP; ^▲^*p* < 0.001 vs. TMP; ^▲▲^*p* < 0.05 vs. TMP. Between the salivary glands: ^#^*p* < 0.001 vs. control in parotid gland; ^*p* < 0.001 vs. CMP in parotid gland; ^○^*p* < 0.001 vs. CMP/R in parotid gland; ^□^*p* < 0.001 vs. TMP in parotid gland; ^◊^*p* < 0.001 vs. TMP/R in parotid gland (Kruskal–Wallis test followed by a Dunn’s multiple comparison post hoc test). *C* control group without any medication; *CMP* rats treated with cyclosporin A, mycophenolate mofetil, and prednisone; *CMP/R* rats treated with cyclosporin A, mycophenolate mofetil, and prednisone in the first 3 months of the experiment and rapamycin in the last 3 months; *PCNA* proliferating cell nuclear antigen; *PRG* parotid gland; *SLG* sublingual gland; *SMG* submandibular gland; *TMP* rats treated with tacrolimus, mycophenolate mofetil, and prednisone; *TMP/R* rats treated with tacrolimus, mycophenolate mofetil, and prednisone in the first 3 months of the experiment and rapamycin in the last 3 months

In the control group and in the experimental groups of animals, immunolocalization of PCNA in the parotid, submandibular and sublingual glands were observed in the form of brown-stained nuclei of acini and ducts (Fig. 5A, Supplementary Fig. 5). Kruskal–Wallis and post hoc Dunn’s tests revealed that in the parotid gland, the percentage of PCNA-positive cells was significantly higher than that in the submandibular gland for the CMP (*H*(2) = 31.97; *p* < 0.001) and CMP/R (*H*(2) = 47.40, *p* < 0.001) groups and was lower than that in the sublingual gland for the control (*H*(2) = 29.27; *p* < 0.001), TMP (*H*(2) = 40.88; *p* < 0.001), and TMP/R (*H*(2) = 41.35; *p* < 0.001) groups. In the sublingual gland, the percentage of these cells was significantly higher (*p* < 0.001, respectively) than that in the submandibular gland for all groups (Fig. 5B).

The percentage of PCNA-positive cells (*n* = 30 in each group) in the parotid gland was significantly higher in the CMP, CMP/R and TMP groups (*H*(4) = 98.55; *p* < 0.001, respectively) than in the control group. In the submandibular gland, the percentage of PCNA-positive cells (*n* = 30 in each group) was significantly higher only in the CMP and TMP groups (*H*(4) = 51.51; *p* < 0.001, respectively) vs. control. In turn, in the sublingual gland, the percentage of these cells (*n* = 30 in each group) was significantly higher in all experimental groups (*H*(4) = 89.79; *p* < 0.001, respectively) groups vs. control.

In all studied glands in the CMP/R and TMP/R groups, the percentage of PCNA-positive cells was significantly lower than that in the CMP (*p* = 0.046, *p* = 0.003, and *p* = 0.001, respectively) and TMP (*p* < 0.001, *p* = 0.002, and *p* = 0.008, respectively) groups (Fig. 5B).

## Discussion

This investigation seems to be important in the context of identifying potential morphological disorders that may translate into abnormal functioning of the salivary glands in patients after organ transplantation. It should be highlighted that this is the first report on the long-term effect of multidrug immunosuppressive regimens based on calcineurin inhibitors and conversion from triple therapy to rapamycin monotherapy on the rat salivary gland.

In this study, a long-term model of immunosuppressive drug administration was developed that can be compared with the therapy used in clinical practice in patients after organ transplantation. The rats were treated for 6 months, which is approximately 15 years of human life, assuming that the average lifespan of a rat is 2–3 years [21]. Unfortunately, in the available literature on the effect of immunosuppressive drugs on the salivary glands in experimental models, these drugs were administered for a relatively short time compared to our experiment [16, 17]. In our study, we intended to determine the direct effect of immunosuppressive drugs on the rat salivary gland. Therefore, the experiment was performed on animals that did not undergo organ transplantation. This made it possible to exclude factors that could interfere with the effect of immunosuppressive drugs, such as the ischemia/reperfusion phenomenon, or immunological factors associated with the activation of cellular and humoral mechanisms of the body in response to transplanted organs. It is worth noting that our experiment used the oral route of drug administration, which represents both the physiological and the safest form. In studies by other authors, animals were often administered drugs subcutaneously [17, 22] or by gastric intubation [16].

### The long-term effects of the conversion of treatment with CNIs to rapamycin in immunosuppressive protocols in the rat salivary glands cause decreased collagen accumulation

In the present study, it was shown that long-term administration of CNIs in multidrug protocols and conversion to rapamycin monotherapy causes morphological changes in the parotid, submandibular and sublingual salivary glands of the rat. In all salivary glands, features of interstitial fibrosis were also found. It should be noted that the FK-506-based regimens showed a lower collagen accumulation (statistically insignificant) than the CsA-based group. Moreover, in the salivary glands of all experimental groups, focal infiltration of inflammatory cells was observed. It is suggested that fibrosis could be the probable effect of chronic infiltration of inflammatory cells, which is in agreement with data reported by Wynn (2008) [23].

The results obtained by other authors seem to confirm our observations, despite the use of CNIs in monotherapy [16, 17]. Spolidorio et al. (2015) have shown alterations of the epithelial architecture in the form of disordered acini, hyperchromasia, evident nucleoli, and atypical mitosis in the parotid and submandibular gland of rats after 60 days of treatment with CsA or FK-506 [17]. Interestingly, these researchers did not observe significant histopathological changes in the salivary glands of rats after 30 days of administration of CNIs, which explains how important it is to observe the long-term effects of the drugs used. In addition, the authors found no significant alterations in pilocarpine-stimulated salivary flow rates due to the treatments with use CNI at any of the studied time-points. However, they observed decreased concentrations of Na^+^, Ca^2+^ and total proteins and also lower activities of antioxidant enzymes after the 60-day treatment with any of the immunosuppressants in the saliva samples collected from parotid and submandibular salivary gland in comparison with the control group. The authors suggest that the prolonged administration of CNIs causes cytological alterations in the salivary glands, which may be related to functional changes. Dehpour et al. (1996) revealed that a 45-day administration of CsA causes both morphological and functional alterations in rat submandibular glands [16]. They observed acinar vacuolation, nuclei with condensed and marginated heterochromatin, mitochondrial alterations, disturbances in cytoplasmic organelles, and a reduction in the number of secretory granules. Moreover authors revealed that in CSA-treated rats Mg^2+^ and K^+^ concentrations were significantly elevated and pilocarpine-stimulated flow rates were reduced to 54% of those in controls. As a consequence, cytological disorders may be related to abnormal concentrations of selected microelements in the tissue. It is worth emphasizing that the authors did not examine fibrosis in the salivary glands. Our research suggests that FK-506-based regimens appear to have less fibrogenic potential than CsA-based regimens. Research performed by Jain et al. (2000) seems to confirm our observations [24]. They revealed that in rats, in a model of renal ischemia–reperfusion injury, FK-506 significantly reduced the expression of transforming growth factor β and tissue inhibitor of metalloproteinases 1, the products of genes associated with fibrosis, compared to expression with CsA administration. As a result, this may translate into the choice of a long-term regimen that will allow the graft to maintain stable function for a longer period.

In our experiment in rats that converted from CNI-based three-drug regimens to rapamycin monotherapy, similar morphological alterations were observed in all salivary glands compared to the animals that were administered CNIs in the three-drug combinations throughout the duration of the experiment. Moreover after the conversion of CsA-based triple therapy to rapamycin, less collagen accumulation was found. In submandibular and sublingual salivary glands, in the FK506-based group converted to rapamycin, tendency to less collagen deposition was also noted compared to scheme without conversion (statistically nonsignificant).

Our results are in agreement with the findings of Soliman et al. [18]. The authors revealed that the administration of rapamycin was associated with morphological changes in the rat submandibular salivary gland. They showed that 3 months of therapy with rapamycin caused marked atrophic and degenerative changes in the form of degeneration of wide areas of the parenchymal and stromal elements and cytoplasmic vacuolization of ductal and acinar cells. Moreover, the dissociation of collagen fibers, degenerated fibroblasts and dilated blood vessels were observed. Similar to the indicated authors, we have demonstrated the antifibrotic effect of rapamycin, which may be of key importance for patients undergoing transplantation of vascularized organs. It might be suggested that conversion of treatment from CNI-based regimens to rapamycin monotherapy exerts beneficial antifibrotic effects on the rat salivary gland through an inhibitory effect on fibroblast growth factor and thus on collagen formation. Interestingly, some available data indicate that rapamycin may have a beneficial effect on the mouse submandibular gland by delaying ligation-induced salivary gland atrophy [22].

Other authors [16, 18] have described more intense morphological changes in the salivary glands of animals after immunosuppressive treatment compared to our observations. These changes might result from the use of daily doses of immunosuppressive drugs that were higher than those applied in our experiment. Dehpour et al. [16] administered CsA at a dose of 25 mg/kg/day, while Spolidorio et al. [17] administered 10 mg/kg/day. In turn, Soliman et al. [18] used rapamycin at a dose of 10 mg/kg/day. It is worth highlighting that we applied doses of immunosuppressants that allowed us to obtain drug concentrations in the blood of the rats within the therapeutic range [19]. High doses of immunosuppressive drugs can reach toxic levels in the blood and can cause more severe side effects in the form of hepatotoxicity, nephrotoxicity or neurotoxicity. Consequently, this toxicity may translate into the failure of many systems and organs, including the salivary glands.

### The long-term effects of conversion of treatment with CNIs to rapamycin in immunosuppressive protocols in the rat salivary glands cause decreased apoptosis and proliferation

In our experiment, in rats treated with CNI-based triple regimens, higher percentages of TUNEL-, Ki67- and PCNA-positive cells were found in the parotid, submandibular and sublingual salivary glands compared to the percentages in the control group. Unfortunately, other authors did not examine the percentage of apoptotic and proliferative cells in salivary glands after immunosuppressive therapy. Only Dehpour et al. [16], using transmission electron microscopy, revealed apoptotic features of acinar cells in the rat submandibular glands after administration of CsA. The researchers observed nuclei with condensed and marginated heterochromatin in the peripheral region clumped along the nuclear membrane. The results obtained in our previous studies on the rat prostate gland [20] seem to be in accordance with the present observations. In the CNI-based groups (where CsA, MMF, and prednisone, as well as FK-506, MMF, and prednisone, had been administered), we found an increased percentage of TUNEL-positive cells in the glandular epithelium and stroma of the ventral prostate compared to the control group. We have also found an increase in the percentage of PCNA-positive cells in both the ventral and dorsal prostate epithelium, but it was statistically nonsignificant [20, 25]. Therefore, it can be assumed that CNI-based regimens may exert proapoptotic and proliferative effects. However, it should be noted that this effect would probably be more pronounced if CNIs were used in monotherapy. The use of CNIs in multidrug combinations might have therapeutic benefits and may be associated with decreased apoptosis or proliferation.

In our experiment in rats that converted from CNI-based three-drug regimens to rapamycin monotherapy, decreased apoptosis and proliferation in all salivary glands were observed compared to the groups without conversion. The antiapoptotic and antiproliferative effects of rapamycin were also noted in our previous studies on the rat prostate [20, 25] and by other authors in different organs [26, 27]. Our research concerning the ventral prostate has also shown a decreased percentage of TUNEL- and PCNA-positive cells both in the ventral and dorsal prostate after conversion of treatment from CNI-based schemes to rapamycin [20, 25]. Yang et al. (2006) revealed that rapamycin significantly decreased apoptosis in the kidneys of rats with induced chronic ischemia/reperfusion [26]. In turn, Zhu et al. confirmed that treatment with rapamycin exerts antiproliferative effects [27]. These authors showed that rapamycin decreased the cell proliferation rate in a human submandibular gland cell line (HSG) following irradiation. Therefore, it may be suggested that conversion of treatment from CNI-based regimens to rapamycin monotherapy exerts beneficial antiapoptotic and antiproliferative effects on the rat salivary gland. The obtained findings may confirm the anticancer effect of rapamycin in the form of decreased cell proliferation and justify the use of mTOR inhibitors in the treatment of cancer patients [28]. It should also be emphasized that immunosuppressive treatment must always be selected individually for the patient and that comorbidities should be taken into account.

The available literature contains data on the analysis of the concentration of immunosuppressive drugs in the saliva of patients. The authors indicate that the measurement of the concentration of immunosuppressive drugs in oral fluids seems to be a feasible and non-invasive method for predicting the concentration of drugs in whole blood [29, 30]. There are also data on the composition of the saliva of patients after organ transplantation, but a number of limitations have been reported [31]. Therefore, further research in this area is justified. Analysis of the composition of the saliva of transplant recipients and the relationship between levels of blood parameters with their salivary levels may help to assess the potential impact of immunosuppressive drugs on the occurrence of particular lesions in the oral cavity.

### Limitations of the study

During the experiment, saliva samples were not collected from the animals. Data concerning protein and electrolyte contents in the saliva would undoubtedly complete the presented results.

Limitations in the use of the rat model should also be taken into account. The results obtained in rodents cannot be directly translated into humans due to interspecies differences, the varied clinical status of patients, and the medications taken by patients. However, despite these limitations, with proper consideration of the interspecies differences when interpreting the results, the obtained findings may have important clinical implications in patients undergoing immunosuppressive therapy.

## Conclusions

Long-term administration of immunosuppressive drugs, both in triple regimens based on CNIs and after conversion to rapamycin monotherapy, causes morphological changes in the salivary glands of rats. Consequently, this may have an effect on salivary glands function.

Immunosuppressive treatment based on CNIs is associated with an increase in collagen accumulation in all salivary glands. However, FK-506-based regimens appear to have less fibrogenic potential than CsA-based regimens, which may be a key element in the selection of a long-term regimen in patients suffering from various diseases.

CNI-based regimens may exert proapoptotic and proliferative effects, while the conversion of treatment from CNI-based regimens to rapamycin monotherapy has antifibrotic, antiapoptotic, and antiproliferative effects on rat salivary glands. These changes may possibly prevent selected abnormalities resulting from the application of CNIs.

### Supplementary Information

Below is the link to the electronic supplementary material.

Supplementary file1 Supplementary Figure 1. Representative light micrographs of hematoxylin and eosin-stained parotid (PRG), submandibular (SMG) and sublingual glands (SLG) of rats in the control, CMP, CMP/R, TMP and TMP/R groups. C − control group without any medication; CMP − rats treated with cyclosporin A, mycophenolate mofetil, and prednisone; CMP/R − rats treated with cyclosporin A, mycophenolate mofetil, and prednisone in the first three months of the experiment and rapamycin in the last three months; PRG – parotid gland; SLG – sublingual gland; SMG – submandibular gland; TMP − rats treated with tacrolimus, mycophenolate mofetil, and prednisone; TMP/R – rats treated with tacrolimus, mycophenolate mofetil, and prednisone in the first three months of the experiment and rapamycin in the last three months. (TIF 73177 KB)

Supplementary file2 Supplementary Figure 2. Representative light micrographs of Masson trichome-stained (blue-stained collagen fibers) parotid (PRG), submandibular (SMG) and sublingual glands (SLG) of rats in the control, CMP, CMP/R, TMP and TMP/R groups. C − control group without any medication; CMP − rats treated with cyclosporin A, mycophenolate mofetil, and prednisone; CMP/R − rats treated with cyclosporin A, mycophenolate mofetil, and prednisone in the first three months of the experiment and rapamycin in the last three months; PRG – parotid gland; SLG – sublingual gland; SMG – submandibular gland; TMP − rats treated with tacrolimus, mycophenolate mofetil, and prednisone; TMP/R – rats treated with tacrolimus, mycophenolate mofetil, and prednisone in the first three months of the experiment and rapamycin in the last three months. (TIF 73177 KB)

Supplementary file3 Supplementary Figure 3. Representative light micrographs of the histochemical reaction showing TUNEL-positive cells in the parotid (PRG), submandibular (SMG) and sublingual glands (SLG) of rats in the control, CMP, CMP/R, TMP and TMP/R groups. C − control group without any medication; CMP − rats treated with cyclosporin A, mycophenolate mofetil, and prednisone; CMP/R − rats treated with cyclosporin A, mycophenolate mofetil, and prednisone in the first three months of the experiment and rapamycin in the last three months; PRG – parotid gland; SLG – sublingual gland; SMG – submandibular gland; TMP − rats treated with tacrolimus, mycophenolate mofetil, and prednisone; TMP/R – rats treated with tacrolimus, mycophenolate mofetil, and prednisone in the first three months of the experiment and rapamycin in the last three months. (TIF 73227 KB)

Supplementary file4 Supplementary Figure 4. Representative light micrographs of the immunohistochemical reaction showing Ki67-positive cells in the parotid (PRG), submandibular (SMG) and sublingual glands (SLG) of rats in the control, CMP, CMP/R, TMP and TMP/R groups. C − control group without any medication; CMP − rats treated with cyclosporin A, mycophenolate mofetil, and prednisone; CMP/R − rats treated with cyclosporin A, mycophenolate mofetil, and prednisone in the first three months of the experiment and rapamycin in the last three months; PRG – parotid gland; SLG – sublingual gland; SMG – submandibular gland; TMP − rats treated with tacrolimus, mycophenolate mofetil, and prednisone; TMP/R – rats treated with tacrolimus, mycophenolate mofetil, and prednisone in the first three months of the experiment and rapamycin in the last three months. (TIF 73177 KB)

Supplementary file5 Supplementary Figure 5. Representative light micrographs of the immunohistochemical reaction showing PCNA-positive cells in the parotid (PRG), submandibular (SMG) and sublingual glands (SLG) of rats in the control, CMP, CMP/R, TMP and TMP/R groups. C − control group without any medication; CMP − rats treated with cyclosporin A, mycophenolate mofetil, and prednisone; CMP/R − rats treated with cyclosporin A, mycophenolate mofetil, and prednisone in the first three months of the experiment and rapamycin in the last three months; PRG – parotid gland; SLG – sublingual gland; SMG – submandibular gland; TMP − rats treated with tacrolimus, mycophenolate mofetil, and prednisone; TMP/R – rats treated with tacrolimus, mycophenolate mofetil, and prednisone in the first three months of the experiment and rapamycin in the last three months. (TIF 73177 KB)

## Data Availability

The datasets generated during and/or analysed during the current study are available from the corresponding author upon reasonable request.

## Abbreviations

C: Control group without any medication
CMP: Rats treated with cyclosporin A, mycophenolate mofetil, and prednisone
CMP/R: Rats treated with cyclosporin A, mycophenolate mofetil, and prednisone in the first 3 months of the experiment and rapamycin in the last 3 months
CNIs: Calcineurin inhibitors
CsA: Cyclosporine A
GD: Granular duct
HE: Hematoxylin and eosin
HSG: Human submandibular gland cell line
mTOR: Mammalian target of rapamycin
PRG: Parotid gland
PBS: Phosphate-buffered saline
PCNA: Proliferating cell nuclear antigen
SD: Striated duct
SLG: Sublingual gland
SMG: Submandibular gland
FK-506: Tacrolimus
TdT: Terminal deoxynucleotidyl transferase
TMP: Rats treated with tacrolimus, mycophenolate mofetil, and prednisone
TMP/R: Rats treated with tacrolimus, mycophenolate mofetil, and prednisone in the first 3 months of the experiment and rapamycin in the last 3 months
TUNEL: Terminal deoxynucleotidyl transferase dUTP nick-end labeling
